# Case Report: Functional Outcome of COVID-19 Subjects With Myasthenia Gravis and Critical Illness Polyneuropathy

**DOI:** 10.3389/fneur.2022.906402

**Published:** 2022-06-21

**Authors:** Domenico Intiso, Antonello Marco Centra, Luigi Amoruso, Michele Gravina, Filomena Di Rienzo

**Affiliations:** ^1^Unit of Neuro-Rehabilitation, IRCCS “Casa Sollievo della Sofferenza”, San Giovanni Rotondo, Italy; ^2^Unit of Neurology, IRCCS “Casa Sollievo della Sofferenza”, San Giovanni Rotondo, Italy

**Keywords:** neurology, myasthenia gravis, COVID-19, ICUAW, neurorehabilitation, outcome

## Abstract

**Background:**

The COVID-19 disease can affect subjects suffering from myasthenia gravis (MG) and worsen its clinical course, leading to intensive care unit (ICU) admission. Critically ill subjects can develop a neuromuscular complication called ICU-acquired weakness (ICUAW). This disorder has also been detected in ICU subjects with COVID-19, but the association between MG and ICUAW has never been described in critically ill patients. We describe the case and functional outcome of a COVID-19 patient suffering from MG who developed critical illness polyneuropathy (CIP).

**Case Presentation:**

A 66-year-old man with a history of hypertension and ocular MG had COVID-19 and required ICU admission. The patient underwent mechanical ventilation and tracheotomy and was treated with remdesivir and corticosteroids. Fifteen days after admission, he complained of tetraparesis without the ocular involvement that remained unchanged despite the increase in anticholinesterase therapy. The length of stay (LOS) in ICU was 35 days. On day 2 of admission, the patient underwent a frontal muscle jitter study that confirmed the MG, and electroneurography (ENG) and electromyography (EMG) that showed overlapping ICUAW with electrophysiological signs characteristic of CIP. The cerebrospinal fluid (CSF) showed normal pressure, cell count, and protein levels (<45 mg/dl) without albumin-cytologic disassociation. The CSF/serum glucose ratio was normal. The CSF culture for possible organisms, laboratory tests for autoimmune disorders, the panel of antiganglioside antibodies, and the paraneoplastic syndrome were negative. Strength and functional outcomes were tested with the MRC scale, the DRS, Barthel scale, and the Functional Independence Measure (FIM) at admission, discharge, and follow-up. Muscular strength improved progressively, and the MRC scale sum-score was 50 at discharge. Anticholinesterase therapy with pyridostigmine at a dosage of 30 mg 3 times daily, which the patient was taking before COVID-19, was resumed. His motor abilities recovered, and functional evaluations showed full recovery at follow-up.

**Conclusion:**

In the described subject, the coexistence of both neuromuscular disorders did not affect the clinical course and recovery, but the question remains about generalization to all patients with MG. The rehabilitation interventions might have facilitated the outcome.

## Introduction

COVID-19 viruses cause characteristic interstitial pneumonia, which causes respiratory failure, but multi-organ systems are also involved, particularly the nervous system, both the central and the peripheral components. Variable conditions and neuropathies affecting the peripheral nervous system (PNS), including Guillain-Barrè syndrome and its variants (1), cranial multifocal neuropathy, dysautonomia (2), and brachial plexus lesion (3), have been described in subjects with COVID-19. Furthermore, neuromuscular disorders such as myalgia, myositis, and, in particular, myasthenia gravis (MG) have also been described (4–6). With regard to the association between MG and the COVID-19 infection, it has been observed that COVID-19 can exacerbate myasthenic crisis (7), promoting worsening of the clinical course and causing severe respiratory failure requiring intensive care unit (ICU) admission (8). It is well-known that, during and after ICU stay, critically ill subjects can develop a neuromuscular complication called ICU-acquired weakness (ICUAW) that embraces a spectrum of disorders including critical illness polyneuropathy (CIP), critical illness myopathy (CIM), and overlapping forms (CIP/CM or CIPNM) (9). The occurrence of ICUAW types in patients with COVID-19 has been reported (10), and this neuromuscular disorder resulted in a common neurological complication in this population during ICU stay. Comorbidities might complicate the course of patients with COVID-19, but concomitant neuromuscular disorders such as MG and ICUAW have not been reported. We have described the case and functional outcome of a man with COVID-19 suffering from MG who developed critical illness polyneuropathy (CIP).

## Case Description

**Figure 1:**
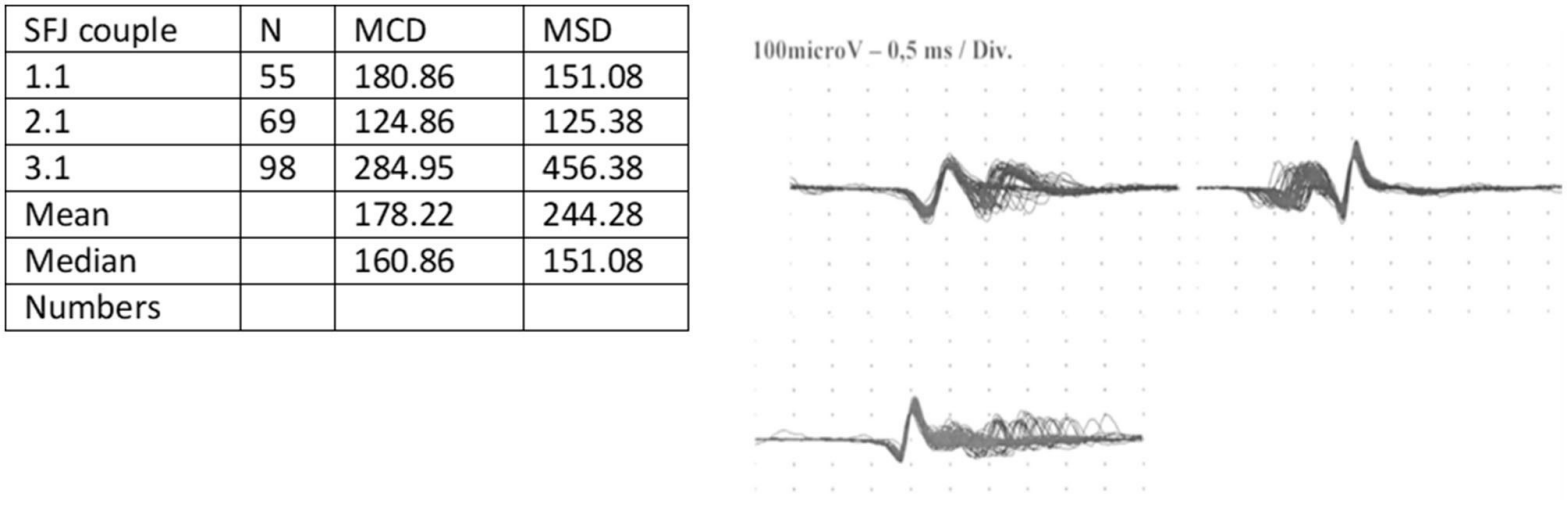
SFEMG jitter by frontal muscle. SFEMG, single fiber electromyography; SFJ, single fiber jitter.

After obtaining the approval of the local ethics committee (Section Giovanni Paolo II- IRCCS Casa Sollievo della Sofferenza) and written informed consent, we report the case of a 66-year-old man with a history of hypertension and ocular MG. This disorder was diagnosed 2 years before the pandemic onset by electromyography (EMG) and frontal muscle jitter study at the neurology unit of our hospital (Figure 1). AChR antibodies were detected, and and the thymoma ascertainment results were negative. He undertook pharmacology therapy consisting of pyridostigmine at a dosage of 30 mg three times daily, which was efficacious in treating myasthenic symptoms. The strength quantification performed by the Medical Research Council (MRC) was normal before the pandemic. At the beginning of December 2020, he developed fever, cough, myalgia, and dyspnea with progressive severe respiratory failure, which required ICU admission. He underwent chest computer tomography (CT) and nasopharyngeal swab that were positive for COVID-19 and was treated with remdesivir and corticosteroids. The patient underwent mechanical ventilation and tracheotomy. Laboratory tests did not detect an increase in the serum CK level. He also developed infections caused by multi-drug resistant germs, including *Klebsiella pneumonia, Acinetobacter baumannii*, and *Pseudomonas aeruginosa*, and underwent multiple antibiotic therapies. The Simplified Acute Physiology Score was 35. During the ICU stay, 15 days after admission, he complained of muscle weakness that evolved into the manifestation of tetraparesis without ocular involvement. Despite this development, pyridostigmine was increased to 60 mg three times daily; the strength remained unchanged. The length of stay (LOS) in ICU was 35 days. After improvement of the clinical conditions, the patient was transferred to our neuro-rehabilitation (NR) unit. At admission, the patient breathed spontaneously but needed 3 L/m oxygen by mask. Capillary oximetry was 97%; he had a central venous catheter, a tracheal tube, and a nasal-gastric tube for nutrition. The neurological picture showed severe tetraparesis that involved predominantly the lower limbs, and he had absent tendinous reflexes. No deficit in ocular or facial muscles was detected, and superficial and deep sensibilities were normal. Given the neurological feature, on day 2 of admission, the patient underwent electroneurography (ENG), electromyography (EMG), and frontal muscle jitter study that confirmed MG and showed overlapping ICUAW. In this respect, the electrophysiological exam revealed signs characteristic of CIP (Table 1). However, a lumbar puncture was performed, and cerebrospinal fluid (CSF) was collected and processed for standard analysis to exclude Guillain-Barrè syndrome and polyneuropathies of different etiology. Pressure, cell count, and protein levels (<45 mg/dl) of the CSF were normal without albumin-cytologic disassociation. CSF/serum glucose ratio was normal. The CSF culture results for possible organisms, such as human immunodeficiency virus, hepatitis B virus, hepatitis C virus, bacteria, Mycobacterium tuberculosis, fungi, Borrelia, enteroviruses, Herpes viruses, and CMV, were negative. Similarly, the laboratory test for autoimmune disorders, including lupus anticoagulant, anticardiolipin antibodies, a panel of antiganglioside antibodies, including anti-GM1, -GM2, -GM3, -GD1a, -GD1b, -GT1b, and -GQ1b, and a panel for paraneoplastic syndrome, were negative.

**Table 1:** Neurophysiological study of patient.

**Nerve**	**Amplitude mV**	**Velocity m/s**
L ulnar motor		
distal	4.6	
proximal	3.2	46.2
R ulnar motor		
distal	4.2	
proximal	3.6	45
L ulnar sensory	-	-
R ulnar sensory	2.3	44
L median motor		
distal	4.5	
proximal	3.7	46
R median motor		
distal	4.2	
proximal	3	45
R median sensory	-	-
L median sensory	1.5	43
Lower Limb		
R peroneal		
distal	<0.2	
proximal	<0.2	35.5
L peroneal		
distal	<0.2	
proximal	<0.2	40
R tibialis		
distal	<0.2	
proximal	<0.2	33
L tibialis		
distal	<0.2	
proximal	<0.2	37.8
Sural		
Right	-	-
Left	-	-

Strength and functional evaluation were quantified through the MRC scale sum-score, the Disability Rating scale (DRS), the Barthel scale (BS), and the functional independence measure (FIM) at admission, discharge, and 6 months of follow-up. During NR stay, the patient underwent a personalized and tailored rehabilitation treatment for 3 h daily, 6 days a week. Furthermore, he performed 2 h of daily electrical muscular stimulation on the lower limbs by placing surface electrodes on the quadriceps and anterior tibial muscles bilaterally. The muscular strength improved progressively, and the MRC sum score was 50 at discharge. The anticholinesterase therapy with pyridostigmine at the dosage of 30 mg three times daily, the same that the patient was taking before he had COVID-19, was resumed. His motor abilities recovered and, at discharge, he was able to walk without support but remained with left foot drop, which required the application of an ankle-foot orthosis (AFO). Furthermore, he complained of mild fatigue with reduced endurance, which improved over time. At follow-up, the MRC scale sum score and all functional scale scores resulted to be normal (Table 2). The LOS in neuro-rehabilitation was 42 days.

**Table 2:** Strength and functional measures scores.

	**EN/EMG**	**MRC**	**BS**	**DRS**	**FIM**
Admission	CIP	26	5	10	48
Discharge		50	95	4	115
Follow-up		60	100	0	126

*CIP, critical illness polyneuropathy; MRC, Medical Research Council scale; BS, Barthel scale; DRS, disability rating scale; FIM, functional independence measure*.

## Discussion

We reported a patient with COVID-19 suffering from MG that developed CIP during the ICU stay. To the best of our knowledge, this is the first case of a subject who had ICUAW that was associated with MG. After rehabilitation, the patient gained back his motor ability, although he required the application of AFO to the left limb and reached full recovery 6 months after discharge. COVID-19 might favor MG. AchR antibody-associated MG (5), MUSK antibody-associated MG (11, 12), and new-onset ocular MG (6) have been reported, but our patient suffered from MG before the pandemic. Viral and bacterial infections, including COVID-19, are established triggers for a myasthenic crisis in patients with preexisting MG (7). Thus, it is conceivable that, in our patient, COVID-19 itself or an exacerbation of myasthenic crisis induced by COVID-19 might have caused respiratory failure, which required ICU admission. The clinical course of patients with MG and COVID-19 can be variable, although the infection does not dramatically influence the course of MG (13). In this respect, long-term chronic corticosteroid treatment, older age, and previously unsatisfactory control of MG symptoms are risk factors for worsening of outcome and high mortality rate in these patients (8, 14). Furthermore, comorbidities are relevant, affecting the clinical course and favoring myasthenic exacerbation, particularly in elder patients with MG (15, 16). In those with MG and COVID-19, comorbidities could be responsible for poor outcomes (17–19). Although our patient developed CIP, he did not have any of the factors, and this condition might explain the favorable outcome of the myasthenic disorder. A recent study has detected a favorable outcome in people with MG vaccinated against COVID-19 and has reported 44% of mortality due to COVID-19 in unvaccinated patients (20). Our patient did not receive any dose of vaccination at the time of COVID-19 contraction because vaccines were not available; nevertheless, he reached full recovery. The impact and effects of anti-COVID vaccination on people with MG are important, but several questions are still unclear. There are no specific guidelines concerning vaccinations of patients with MG, and several doubts remain unsolved, including efficacy, timing and type of vaccine, and risk of exacerbation of MG after COVID-19 (21). Regarding the occurrence of ICUAW in this subject, several hypotheses can be made. ICUAW is a common neurological complication in ICU patients, and a median prevalence of 43% has been reported (22). Therefore, ICUAW could occur regardless of clinical conditions that require ICU admission. In this respect, ICUAW has been frequently detected in critically ill subjects with COVID-19 who experience a severe inflammatory condition (23). The same risk factors and the severity of the systemic disease itself might favor the occurrence of ICUAW in subjects with COVID-19. Therefore, unsurprisingly, our patient with MG developed ICUAW. This disorder can produce severe impairment and persistent disability with poor quality of life. Concerning that point, although several questions remain unsolved about the rehabilitative strategies to carry out on these subjects, a recent systematic review has detected that 70.3% of ICU survivors with ICUAW could achieve a good recovery (24). However, the outcome in patients with COVID-19 who developed ICUAW remains uncertain. We have recently described the clinical course and functional outcome of four patients with COVID-19 and ICUAW, and, at the same time, we performed a review of the literature on this issue. Regarding the functional outcome, the percentage of subjects with COVID-19 and ICUAW who gained good recovery was lower than that of general patients with ICUAW. On the other hand, we observed that the functional outcome in our subjects with COVID-19 and ICUAW was in line with the findings reported in the literature. Indeed, three out of four subjects (75%) reached full recovery. Several reasons may explain this contrasting finding: one could be the measurements used for the functional evaluation and another one could be that ICU specialists may have preferred to describe this neurological complication in patients with COVID-19 during the ICU stay or at discharge and overlooked to report the recovery. However, in particular, the main reason could be that the rehabilitative treatment may have had a role in producing the reported benefits and in improving the outcome. The COVID pandemic has significantly influenced the outcome in patients affected by neuromuscular disease, including patients with MG with relevant consequences on the quality of life, leading to an increase in sedentary behavior and a related decrease in the practice of physical activity (25). In this respect, the patient described in this report underwent a tailored rehabilitation program, and the rehabilitative interventions might be the reason for the observed benefit during recovery.

Some limitations should be considered. This case report concerned “ocular MG” and not the generalized type that might present a different course. Indeed, previous studies have reported worse outcomes in patients with generalized MG and COVID-19. Therefore, the present finding cannot be extended to different types of MG. Furthermore, subtype (ocular versus generalized), serotype, and immunosuppression are important factors to be taken into account. The AChR-MG subtype presents a different response to immunomodulatory regimens compared to antiMuSK (26), and the differences might reflect a different behavior after the COVID-19 infection.

Another factor to take into account when considering COVID-19 is the possible direct damage caused by the virus on the nerves and muscles. Even without evidence of viral invasion of the nervous system, immune-mediated events, through either the cytokine or the chemokine pathway, may lead to tissue and organ damage (27). Therefore, it is conceivable that the same pathological conditions that affect critically ill subjects characterize COVID-19 subjects and may represent a milieu that favors ICUAW.

## Conclusion

The association between MG and ICUAW has never been described in patients with COVID-19 so far. Although in the present subject, the coexistence of both neuromuscular disorders did not affect the clinical course and recovery, unsolved questions remain about generalization to all patients with MG. The rehabilitation intervention might have facilitated the outcome.

## Data Availability Statement

The original contributions presented in the study are included in the article/supplementary material, further inquiries can be directed to the corresponding author/s.

## Ethics Statement

The studies involving human participants were reviewed and approved by local Ethics Committee (Section Giovanni Paolo II- IRCCS Casa Sollievo della Sofferenza). The patients/participants provided their written informed consent to participate in this study.

## Author Contributions

DI and FR: conceptualization. AC, MG, and LA: data extraction. DI, AC, and FR: manuscript preparation. DI, MG, and FR: review and revision. All authors contributed to the article and approved the submitted version.

## Conflict of Interest

The authors declare that the research was conducted in the absence of any commercial or financial relationships that could be construed as a potential conflict of interest.

## Publisher's Note

All claims expressed in this article are solely those of the authors and do not necessarily represent those of their affiliated organizations, or those of the publisher, the editors and the reviewers. Any product that may be evaluated in this article, or claim that may be made by its manufacturer, is not guaranteed or endorsed by the publisher.
